# Metastasis directed stereotactic radiotherapy in NSCLC patients progressing under targeted- or immunotherapy: efficacy and safety reporting from the ‘TOaSTT’ database

**DOI:** 10.1186/s13014-020-01730-0

**Published:** 2021-01-06

**Authors:** Stephanie G. C. Kroeze, Jana Schaule, Corinna Fritz, David Kaul, Oliver Blanck, Klaus H. Kahl, Falk Roeder, Shankar Siva, Joost J. C. Verhoeff, Sonja Adebahr, Markus M. Schymalla, Markus Glatzer, Marcella Szuecs, Michael Geier, Georgios Skazikis, Irina Sackerer, Fabian Lohaus, Franziska Eckert, Matthias Guckenberger

**Affiliations:** 1 Department of Radiation Oncology, University Hospital Zürich, Rämistrasse 100, 8091 Zürich, Switzerland; 2 Department of Radiation Oncology, Marienhospital Stuttgart, Böheimstrasse 37, 70199 Stuttgart, Germany; 3 Department of Radiation Oncology, Charité-University Hospital Berlin, Charitestraße 1, 10117 Berlin, Germany; 4 Department of Radiation Oncology, University Medical Center Schleswig-Holstein, Arnold-Heller-Straße 3, Haus 50, 24105 Kiel, Germany; 5 Department of Radiation Oncology, University Clinic Augsburg, Stenglinstraße 2, 86156 Augsburg, Germany; 6 Department of Radiation Oncology, University Hospital Munich, Georgenstraße 5, 80799 Munich, Germany; 7 Department Or Radiation Oncology, Peter MacCallum Cancer Centre, 305 Grattan St, Melbourne, VIC 3000 Australia; 8 Department of Radiation Oncology, University Medical Center Utrecht, Heidelberglaan 100, 3584 CX Utrecht, The Netherlands; 9 Department of Radiation Oncology, Medical Center, Faculty of Medicine, University of Freiburg and German Cancer Consortium (DKTK), German Cancer Research Center (DKFZ), Partner Site Freiburg, German Cancer Research Center (DKFZ), Heidelberg, Germany; 10 Department of Radiation Oncology, Philipps-University Marburg, Baldingerstraße, 35043 Marburg, Germany; 11 Department of Radiation Oncology, Saint Gallen Cantonal Hospital, Rorschacher Str. 95/Haus 03, 9007 St. Gallen, Switzerland; 12 Department of Radiation Oncology, University Hospital Rostock, Südring 75, 18059 Rostock, Germany; 13 Department of Radiation Oncology, Ordensklinikum Linz, Fadingerstraße 1, 4020 Linz, Austria; 14 Department of Radiation Oncology, Schwarzwald-Baar Klinikum, Klinikstraße 11, 78050 Villingen-Schwenningen, Germany; 15 Department of Radiation Oncology, Strahlentherapie Freising Und Dachau, Biberstraße 15, 85354 Freising, Germany; 16 Department of Radiation Oncology, University Hospital Dresden, Händelallee 28, 01309 Dresden, Germany; 17 Department of Radiation Oncology, University Hospital Tübingen, Hoppe-Seyler-Str. 3, 72076 Tübingen, Germany

**Keywords:** Stereotactic, Radiotherapy, Immunotherapy, Targeted therapy, Concurrent, Oligometastases, NSCLC

## Abstract

**Background:**

Metastasis directed treatment (MDT) is increasingly performed with the attempt to improve outcome in non-small cell lung cancer (NSCLC) patients receiving targeted- or immunotherapy (TT/IT). This study aimed to assess the safety and efficacy of metastasis directed stereotactic radiotherapy (SRT) concurrent to TT/IT in NSCLC patients.

**Methods:**

A retrospective multicenter cohort of stage IV NSCLC patients treated with TT/IT and concurrent (≤ 30 days) MDT was established. 56% and 44% of patients were treated for oligoprogressive disease (OPD) or polyprogressive disease (PPD) under TT/IT, polyprogressive respectively. Survival was analyzed using Kaplan–Meier and log rank testing. Toxicity was scored using CTCAE v4.03 criteria. Predictive factors for overall survival (OS), progression free survival (PFS) and time to therapy switch (TTS) were analyzed with uni- and multivariate analysis.

**Results:**

MDT of 192 lesions in 108 patients was performed between 07/2009 and 05/2018. Concurrent TT/IT consisted of EGFR/ALK-inhibitors (60%), immune checkpoint inhibitors (31%), VEGF-antibodies (8%) and PARP-inhibitors (1%). 2y-OS was 51% for OPD and 25% for PPD. After 1 year, 58% of OPD and 39% of PPD patients remained on the same TT/IT. Second progression after MDT was oligometastatic (≤ 5 lesions) in 59% of patients. Severe acute and late toxicity was observed in 5.5% and 1.9% of patients. In multivariate analysis, OS was influenced by the clinical metastatic status (p = 0.002, HR 2.03, 95% CI 1.30–3.17). PFS was better in patients receiving their first line of systemic treatment (p = 0.033, HR 1.7, 95% CI 1.05–2.77) and with only one metastases-affected organ (p = 0.023, HR 2.04, 95% CI 1.10–3.79). TTS was 6 months longer in patients with one metastases-affected organ (p = 0.031, HR 2.53, 95% CI 1.09–5.89). Death was never therapy-related.

**Conclusions:**

Metastases-directed SRT in NSCLC patients can be safely performed concurrent to TT/IT with a low risk of severe toxicity. To find the ideal sequence of the available multidisciplinary treatment options for NSCLC and determine what patients will benefit most, a further evaluated in a broader context within prospective clinical trials is needed continuation of TT/IT beyond progression combined with MDT for progressive lesions appears promising but requires prospective evaluation.

*Trial registration*: retrospectively registered

## Introduction

The development of targeted-, and immunotherapy (TT/IT) has improved the prognosis of stage IV non-small cell lung cancer (NSCLC) significantly. However, due to the development of resistance, most patients will eventually develop progressive disease. Since stereotactic radiotherapy (SRT) has emerged as a safe and locally effective modality for treatment of oligometastases, its use as part of the multimodality treatment in this situation is increasing [1], as radical local radiotherapy of persistent or progressive (oligo-) metastases could improve outcome [2–6].

Three randomized trials have shown that addition of a localized metastases-directed treatment (MDT) to systemic therapy improved progression free survival (PFS) as well as overall survival in NSCLC patients with oligometastatic disease [7–9]. However, the definition of oligometastatic disease varies in literature, while research on the identification of a biological oligometastatic state is ongoing [10]. This becomes obvious in a systematic review on oligometastatic NSCLC patients, where a variation of 5-year OS between 8.3% and 86% was observed [11]. The subcategorization of (oligo-)metastatic patients remains a challenge, but is important as the value of local treatments varies between these patients [3, 12].

Besides oligometastatic disease, SRT might also play a role as “salvage” treatment in the management of patients who have (oligo-)progressive metastatic disease while under TT/IT [12]. Once a stable oncological status is achieved under systematic therapy and no severe side effects develop, a patient preferably continues this drug for as long as possible, assuming that the further lines of systematic treatment are characterized by a worse therapeutic ratio. Unfortunately, intrinsic or acquired resistance to systematic drugs develops in nearly all patients [13]. Here, MDT could possibly prevent or delay the switch of systematic therapy by radical local treatment of all progressive metastatic sites. This study aimed to evaluate efficacy and safety of metastasis directed SRT (MDT) in NSCLC patients who are progressive under immuno- or targeted therapy.

## Materials and methods

A retrospective international multicenter registry study was established by the German Society for Radiation Oncology (DEGRO) working group for radiosurgery and stereotactic radiotherapy to collect data on stage IV patients receiving SRT with concurrent targeted- or immunotherapy (TOaSTT study). The study was approved by the ethics committees at all participating sites (BASEC-Nr. 2016–01807). For this study, all patients with NSCLC were evaluated. Patients were treated with SRT between 07/2009 and 05/2018. Inclusion criteria were: ≥ 18 years of age, diagnosis of stage IV synchronous or metachronous metastatic disease, histological confirmation of NSCLC, SRT of any cranial or extracranial local recurrence or metastasis, treated concurrently (≤ 30 days) with any type of following systemic treatments: antibodies, tyrosine kinase inhibitors and/or immune-checkpoint inhibitors. Cranial SRT was defined as delivery of up to 5 fractions, or one fraction with a minimum of 16 Gy. Stereotactic body radiotherapy (SBRT) was defined as delivery of ≤ 10 fractions with a minimum total dose of 50 Gy (2 Gy equivalent, α/β of 10 Gy; the α/β-ratio of 10 represents the intrinsic radiosensitivity of NSCLC metastases [14]).

To further evaluate clinical scenarios in which MDT is currently often performed, the group of oligoprogressive disease patients (OPD, ≤ 5 metastases) and polyprogressive disease patients (PPD, > 5 metastases) was sub-analyzed. The OPD cohort consisted of patients where MDT of either all metastases or all oligoprogressive metastases was performed; the PPD cohort was characterized by patients, where polyprogressive disease was observed and only dominant lesions were treated with MDT, according to local interdisciplinary decision. Endpoints were overall survival (OS), time to therapy switch (TTS), progression free survival (PFS), local metastases control (LC), and toxicity. OS was defined as time of SRT to time of death, living patients were censored at the date of last follow-up. PFS and LC were defined as time of SRT to time of progression, which was determined by PET-CT/MRI, MRI, CT, ultrasound or X-ray imaging. PFS and LC were evaluated by censoring patients at their most recent imaging. TTS was defined as time of MDT to time of start of a new systemic therapy. Acute severe toxicity (grade ≥ 3 events, < 3 months after SRT) probably caused by MDT was analyzed using the Common Terminology Criteria for Adverse Events (CTCAE) v4.03. Late severe (grade ≥ 3 events) toxicity was evaluated in patients with a follow-up of ≥ 3 months.

Descriptive statistical analysis was performed with SPSS v25.0 statistic software package (IBM Corp., Armonk, NY, USA). Kaplan–Meier survival curves with log-rank analysis for comparison of subgroups was used to evaluate OS, PFS, and TTS. The Fisher`s exact and Chi-square test was used to compare differences between two independent groups. Univariate and backward multivariate Cox regression analysis was performed to identify independent variables for OS, PFS and TTS. A p-value of less than 0.05 was deemed statistical significant.

## Results

### Patient characteristics

Data of 108 patients from 16 participating centers was included. Baseline patient, tumor and treatment characteristics are summarized in Table 1. Median patient age was 63 (range 33–80) years, 90% of patients had an ECOG performance score of ≤ 1, 41% had minimal co-morbidities (age-adjusted Charlson Comorbidity Index ≤ 3). The most frequent histological NSCLC subtype was adenocarcinoma (80%). Multiorgan metastatic disease was present in 71% of patients, with a median of 2 (range 1–7) involved organs per patient. Reported reasons for MDT in PPD patients were palliation of symptoms (26%), prevention of future complications (79%), attempt to extend treatment with current systemic therapy (11%) and to induce a possible immunomodulation effect (9%).

**Table 1:** Patient characteristics of all 108 NSCLC patients treated with SRT concurrent to TT/IT

	N (%), median (range)
	All patients (n = 108)
Age (years)	63 (33–80)
Histology subtype	
ADC	80 (74)
LCNEC	3 (3)
SqCC	6 (6)
Adenosquamous	3 (3)
Unknown	16 (15)
Synchronous metastatic disease	
Yes	81 (75)
No	27 (25)
Ligand expression/driver mutation	
EGFR	49 (45)
ALK	16 (15)
ROS1	2 (2)
PD-L1	5 (5)
No	30 (28)
Unknown	5 (5)
Previous systemic treatment lines	1 (1–5)
Present metastases	
≤ 5	53 (49)
> 5	55 (51)
Involved organs	2 (1–7)
Status of primary tumor	
Controlled	75 (69)
Progressive	26 (24)
Unknown	7 (7)
SRT treated lesions	
Brain	144 (75)
Lymph nodes	3 (2)
Lung	18 (9)
Liver	6 (3)
Adrenal gland	3 (2)
Bone	17 (9)
Soft tissue	1 (0.5)
SRT treated lesions per patient	
Cranial	1 (1–5)
Extracranial	1 (1–3)
Type of systemic therapy	
EGFR/ALK-inhibitor	65 (60)
PD-1/PD-L1-inhibitor	33 (31)
Anti-VEGF-antibody	9 (8)
PARP-inhibitor	1 (1)
Prescribed BED_10_ (Gy)	
Cranial	75 (26.6–113.9)
Extracranial	95.3 (53.1–180)
Total GTV volume (cc)	
Cranial	1.2 (0.04–15.3)
Extracranial	8.4 (0.5–86.1)

*ADC *adenocarcinoma, *LCNEC * large cell neuroendocrine carcinoma, *SqCC *squamous cell carcinoma

### Targeted therapy/immunotherapy

Overall, 56% of patients received first line systemic therapy, while 44% were under second-line therapy or more (Table 1). Systemic therapy concurrent to MDT consisted of EGFR/ALK-inhibitors (60%), immune checkpoint inhibitors (31%), VEGF-antibodies (8%) and PARP-inhibitors (1%). In 10% of patients, these were combined with chemotherapy. Sixty-seven percent of patient started TT/IT before MDT, with a median of 269 days (range 1–180 days), 8% started IT/TT at the same time of SRT and 25% started IT/TT a median of 14 days (range 1–30) after SRT. Overall, in 28% of patients their systemic therapy was paused during MDT with a median of 10 (range 2–42) days. Targeted therapy was paused in 35% of the patients, for a median of 3 days before and 3 days after MDT (range 1–21 days). Immune checkpoint inhibitors were paused in 15% of patients, for a median of 7 days before and after MDT (range 1–19 days). Bevacizumab was paused in 20% of patients for a median of 14 days before up to 14 days after MDT (range 7–21 days). The decision to pausing TT/IT as well as the length of the TT/IT interruption during SRT was at the discretion of the participating center.

### Stereotactic radiotherapy

In total, 192 lesions were irradiated. Brain metastases were the most frequent location (68%), with a median number of 1 (range 1–5) brain metastasis treated per patient. Median total tumor volume of brain metastases was 1.2 cc (range 0.04–15.3). Median prescribed dose for brain metastases was 20 Gy in 1 fraction (BED_10_ = 75 Gy). SBRT was performed in 32% of patients, PPD patients received SBRT less often compared to OPD patients (21% vs. 41% respectively). A median of 1 (range 1–3) extracranial metastases were treated simultaneously per patient. Extracranial metastases were treated with a median dose of 95.3 Gy (BED_10_) in median 3 fractions (range 1–5). Median planning targed volume of SBRT was 8.37 cc (range 0.54–86.10 cc).

### Efficacy and factors influencing survival

**Fig. 1:**
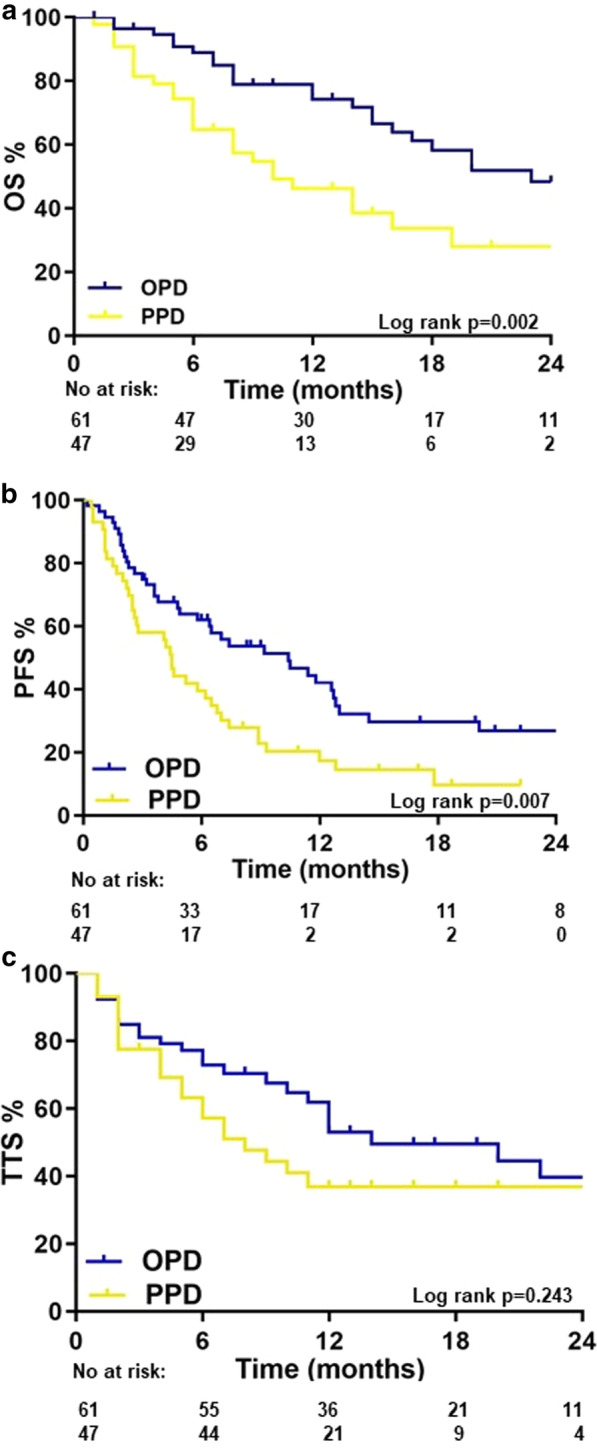
**a** Overall survival of metastatic NSCLC patients receiving metastasis directed therapy (MDT) concurrent to targeted- or immunotherapy (TT/IT). **b** Progression free survival (PFS) of oligoprogressive disease (OPD) NSCLC patients receiving MDT concurrent to targeted- or immunotherapy (TT/IT). **c** Time to systemic therapy change (TTS) after MDT in in NSCLC patients receiving concurrent SRT and TT/IT. Blue line = OPD patients where a MDT of all present metastases (≤ 5 metastases) was performed. Yellow line = OPD patients where MDT of all progressive lesions was performed and all other metastases are controlled by TT/IT

Median follow-up was 18.7 (range 1–102) months. Median OS was 18.1 months, 2 year OS was 39% (Fig. 1). Cause of death was tumor-related in 85% and never therapy-related. There was no significant difference in OS between patients with- or without brain metastases (p = 0.181). There was also no significant difference in OS between patients receiving IT, TT, AAT or PARPi (p = 0.765). In univariate analysis, metastatic status (OPD vs. PPD) and number of affected organs were significant predictors of OS (Table 2). In multivariate analysis, metastatic status remained the only independent factor predicting OS (p = 0.002, HR 2.03 (95% CI; 1.3–3.17). Local control after SRT was 84% after 2 years, there was no difference between OPD and PPD. Overall, median PFS was 8.7 months. In OPD, this was median 10.4 months and 7.4 months for PPD patients, or 25% and 8% after 2 years, respectively (Fig. 1). In the univariate analysis, metastatic status, previous lines of systemic therapy and affected organs were significant predictors of PFS. In multivariate analysis, the number of previous lines of systemic therapy [p = 0.033, HR 1.7 (95%CI 1.05–2.77)] and number of affected organs (p = 0.023, HR 2.04 (95% CI 1.10–3.79) remained independent factors predicting PFS (Table 2).

**Table 2:** Uni- and multivariate Cox regression analysis

Variables	OS			PFS			TTS		
	Univariate	Multivariate		Univariate	Multivariate		Univariate	Multivariate	
	p value	p value	HR (95% CI)	p value	p value	HR (95% CI)	p value	p value	HR (95% CI)
Cinical metastatic status (OPD, palliative intent)	0.006	0.002	2.03 (1.30–3.17)	0.002	0.334		0.053	0.938	1.0 (0.56–1.81)
Initial stage (I–IV)	0.567	–	–	0.402			0.561		
Metastatic development (synchronous vs. metachronous)	0.802	–	–	0.895			0.653		
Previous lines of systemic treatment (1 vs. > 1)	0.166	0.109	–	0.048	0.033	1.7 (1.05–2.77)	0.093	0.152	1.57 (0.84–2.92)
Metastatic burden (1, 2–5, 6–10, > 10)	0.461	–	–	0.062	0.759		0.051	0.686	1.10 (0.741.64)
Affected organs (1 vs. > 1)	0.03	0.633	–	0.004	0.023	2.04 (1.10–3.79)	0.026	0.031	2.53 (1.09–5.89)
Location of metastases (cranial vs. extracranial)	0.201	0.827	–	0.155	0.757		0.48		
Histology subtype (SqCC, ADC, LCNEC, adenosquamous, unknown)	0.952	–	–	0.554			0.447		
Gene mutation (yes vs. no)	0.834	–	–	0.992			0.696		
Status of primary tumor (controlled vs. progressive)	0.973	–	–	0.701			0.824		
Targeted therapy (IT, TT, AAT)	0.855	–	–	0.424			0.64		
SRT location (cranial, extracranial, both)	0.25	–	–	0.149	0.571		0.767		
Number of SRT treated metastases (1–5)	0.642	–	–	0.993			0.509		
Start targeted therapy (before, during, after SRT)	0.239	–	–	0.436			0.299		
Targeted therapy paused during SRT (yes vs no)	0.645	–	–	0.734			0.104	0.393	1.32 (0.75–2.34)

p < 0.05 is significant

*HR * hazard ratio, *CI *confidence interval, *TT *targeted therapy, *IT *immunotherapy, *AAT *antiangiogenic therapy, *PARPi * PARP-inhibitor

### Patterns of disease progression

In case of progression, only one organ was affected (range 1–4) in 69% of OPD patients and 36% of PPD patients. These recurrences were oligometastatic (≤ 5 new lesions) in 59% of OPD patients, with no difference between concurrent systemic therapy (p = 0.765). Forty-three percent of patients that developed a new oligoprogression received a second course of MDT. Other local therapies consisted of surgery in 2 patients and conventionally fractionated radiotherapy in 5 patients.

**Fig. 2:**
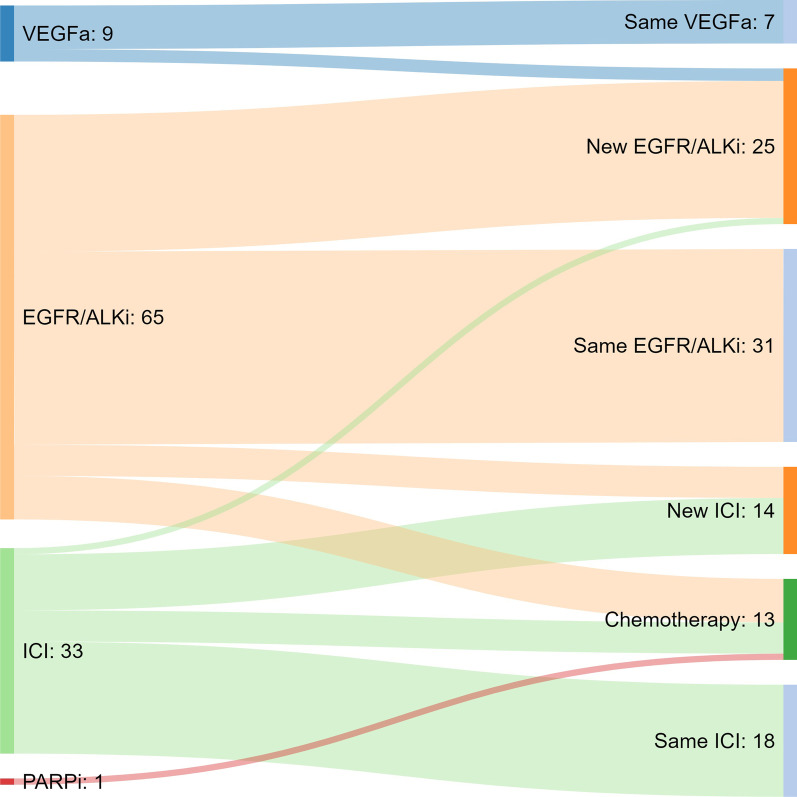
Flow chart of systemic therapy switch following SRT

After one year, 58% of OPD patients and 39% of PPD patients were receiving the same systemic therapy as at the time of MDT. The median TTS was 14 months (range 5.7–22.3 months) for OPD patients and 8 months for PPD patients. There was no significant difference between administered systemic therapy (p = 0.220). In patients where systemic treatment was switched, the next line of treatment was usually a new targeted therapy in OPD patients (68%) and chemotherapy in PPD patients (52%) (Fig. 2). Patients receiving IT who had progressive disease after SRT switched to another IT (60%) or chemotherapy (33%), patients receiving TT who developed progressive disease after SRT most commonly switched to another TT (65%) (Fig. 2).

### Toxicity

Acute severe (≥ grade 3, < 30 days) toxicity likely caused or worsened by MDT was observed in 6 patients (5.5%), consisting of 7 grade 3 toxicities and 1 grade 4 toxicity (Table 3). Late severe toxicity (≥ grade 3, ≥ 30 days) was observed in 2 patients (1.9%); one patient with two 3 grade toxicities and 1 grade 4 toxicity, and one patient with grade 3 weight loss. Most severe toxicities occurred after SRT of brain metastases and in two cases after SBRT of lung metastases. All severe toxicities occurred in patients receiving immune checkpoint inhibition, VEGF-antibody or EGFR-inhibitors. There was no clear pattern of occurrence of severe toxicity observed (Table 3). No grade 5 toxicity occurred.

**Table 3:** Observed infield acute and late severe (≥ grade 3) toxicity, graded by use of CTCAE v4.03

Patient (n)	Toxicity	Grade	Location SRT	Treated metastases (n)	Treatment dose (Gy/fx)	Concurrent therapy	Start of concurrent therapy	Systemic therapy paused during SRT
Acute infield toxicity								
1	Headache	3	Brain	3	24 Gy/1fx 100% Isodose)	Nivolumab	8 days after SRT	–
2	Headache	3	Brain	1	20 Gy/1fx (80% Isodose)	Bevacizumab	365 days before SRT	No
3	Headache	3	Brain	2	20 Gy/1fx (80% Isodose)	Nivolumab	52 days before SRT	No
	Gait disturbance	3						
4	Headache	4	Brain	3	20 Gy/1fx (80% Isodose)	Nivolumab	11 days after SRT	–
	Nausea	3						
5	Dyspnea	3	Lung	1	7 Gy/5fx (65% Isodose)	Gefitinib	503 days before SRT	No
6	Thromboembolic event	3	Brain	1	20 Gy/1fx (80% Isodose)	Osimertinib	98 days before SRT	No
Late infield toxicity								
7	Radionecrosis	3	Brain	5	20 Gy/1fx (80% Isodose)	Afatinib	22 days before SRT	No
	Nausea	3						
	Hemiparesis	4						
8	Weight loss	3	Lung	1	7 Gy/5fx (65% Isodose)	Erlotinib	575 days before SRT	4 days

## Discussion

This analysis of stage IV NSCLC patients receiving MDT for progressive or persistent metastases under targeted-, or immunotherapy showed survival rates in the OPD group that appear promising compared to literature on patients receiving TT/IT alone [15–17]. The concept of targeting OPD while continuing systemic therapy beyond progression is increasingly performed [18, 19]. This is based on the observation that (1) further lines of systematic treatment are characterized by a worse therapeutic effect, (2) MDT obtains good results with limited toxicity in the primary oligometastatic situation [7–9], and (3) whole genomic sequencing studies showing that through parallel evolution, subpopulations of metastatic clones are capable to metastasize themselves [20]. This may indicate that MDT of these lesions could possibly improve prognosis. Literature on the concept of MDT for OPD is still limited and includes, next to small retrospective studies [2–6], a phase II study, which showed that MDT of ≤ 6 progressive metastases in platinum-refractory NSCLC patients who received erlotinib resulted in a better PFS and OS than would be expected in patients receiving TT alone [21]. In all available studies, metastatic patients in varying phases of their treatment were included, which increases the difficulty to interpret their results and transfer the data to clinical practice. Furthermore, the increasing use of immunotherapy in this population has so far not been taken into account. Our study therefore analyzes real-life data of the efficacy and safety of MDT performed in metastatic NSCLC patients progressive under targeted-, as well as immunotherapy.

MDT resulted in a good OS in OPD patients, and allowed continuation of systemic therapy within the first year for many patients. The effect of MDT in the patient group treated with palliative intent was less pronounced in terms of OS and PFS. However, these patients were most frequently treated with SRT for brain metastases. Some TT/IT are characterized by good penetration of the blood–brain barrier; however, upfront MDT of cerebral metastases might improve patient outcome and quality of life [22–25].

When a new progression occurred after MDT, this was often again OPD and a repeat-irradiation was performed in most of these patients. The effectiveness of repeat-irradiations has been previously published for prostate cancer recurrences and treatment of brain metastases, generally resulting in an excellent local control with limited toxicity [22]. In our multivariate analysis, it was shown that the number of lines of systemic therapy influenced PFS. This reflects that the reducing efficacy of subsequent lines of therapy drives distant progression and appears to be more important than metastatic burden at time of MDT. Repeat-MDT instead of therapy-switch may therefore play an increasingly important role. However, experiences of repeat-irradiation remain limited and further studies need to investigate carefully the concept of repeat local treatment.

Although the concept of MDT for OPD under TT/IT is practiced with increasing frequency, several uncertainties remain. First of all, with the currently limited knowledge on the molecular background of resistant clones in NSCLC, it is not known whether preferably all metastases or just selected progressive metastases should be targeted, and what the threshold of the number and volume of targeted metastases should be [26]. Current decision-making is based on imaging and clinical criteria [11, 27], but studies on the biological status of metastatic NSCLC is urgently needed, including integration of liquid biopsy for staging and response assessment. Secondly, the timing of MDT currently remains a clinical decision, usually based on CT or PET-images. However, progression under TT/IT can be very slow, or after immunotherapy, a pseudoprogression could be observed. Thirdly, there might be different strategies in combining systemic therapies with MDT, as the effects of concurrent treatment may be diverse. For example, combining immunotherapy with MDT could possibly strengthen the antitumor immune response [28], whereas abscopal effects of radiotherapy are exceedingly unlikely in patients not receiving immunotherapy [26].

A limitation of this study is its retrospective nature which, however, is a way of a meaningful evaluation of a quickly changing clinical field and associated limitations in standardization of reporting of factors, such as toxicity and local control. Since there is a known risk of underreporting low grade toxicity in retrospective studies, only high grade toxicity was registered [29]. Furthermore, as all patients received a combined modality treatment, our study does not allow to evaluate the influence of the continued TT/IT alone on outcome. It may be possible, that patients with less metastatic sites have had a better prognosis irrespective of MDT. Especially under immunotherapy, residual sites potentially could remain stable for a longer time. However, for patients receiving targeted therapy a further progress of residual sites can be expected after several months. A first study comparing targeted therapy as monotherapy to a combined modality therapy with SRT indicated a benefit of combined therapy compared to targeted drugs alone [30] and will be further investigated in the randomized HALT (NCT03256981) and STOP-NSCLC NCT02756793 trials. Another limitation is the combined analysis of patients treated with IT/TT in one study population. This was done because the intention of MDT was similar irrespective of the systemic therapy, namely ablation of (oligo-) progressive metastases while the otherwise effective systemic therapy is continued.

In conclusion, metastases-directed SRT in NSCLC patients can be safely performed concurrent to TT/IT with a low risk of severe toxicity. To find the ideal sequence of the available multidisciplinary treatment options for NSCLC and determine what patients will benefit most, a further evaluated in a broader context within prospective clinical trials is needed continuation of TT/IT beyond progression combined with MDT for progressive lesions appears promising but requires prospective evaluation.

## Data Availability

The datasets used and/or analysed during the current study are available from the corresponding author on reasonable request.

## Abbreviations

AAT: Antiangiogenic therapy
BED: Biologically effective dose
CTCAE: Common terminology criteria for adverse events
EGFRi: Epidermal growth factor receptor inhibitor
IT: Immunotherapy
MDT: Metastasis directed treatment
NSCLC: Non small cell lung cancer
OPD: Oligoprogressive disease
PARPi: Poly(ADP-ribose)-polymerasen inhibitor
PPD: Polyprogressive disease
SBRT: Stereotactic body radiotherapy
SRT: Stereotactic radiotherapy
TT: Targeted therapy
VEGFa: Vascular endothelial growth factor antibody
